# Identification of Bioactive Substances Derived from the Probiotic-Induced Bioconversion of *Lagerstroemia speciosa* Pers. Leaf Extract That Have Beneficial Effects on Diabetes and Obesity

**DOI:** 10.3390/microorganisms12091848

**Published:** 2024-09-06

**Authors:** Byung Chull An, Sang Hee Kwak, Jun Young Ahn, Hye Yeon Won, Tae Hoon Kim, Yongku Ryu, Myung Jun Chung

**Affiliations:** 1 R&D Center, Cell Biotech Co., Ltd., Gimpo-si 10003, Republic of Korea; bcan@cellbiotech.com (B.C.A.); shkwak@cellbiotech.com (S.H.K.); jyahn@cellbiotech.com (J.Y.A.); hywon@cellbiotech.com (H.Y.W.); ykryu@cellbiotech.com (Y.R.); 2 Department of Food Science and Biotechnology, Daegu University, Gyeongsan 38453, Republic of Korea; skyey7@daegu.ac.kr

**Keywords:** *Lactobacillus plantarum* CBT-LP3, probiotic, bioconversion, metabolomics, anti-diabetic activity, *Lagerstroemia speciosa* L. (Banaba)

## Abstract

*Lagerstroemia speciosa* L. (Banaba) has been used as a functional food because of its diuretic, decongestant, antipyretic, anti-hyperglycemic, and anti-adipogenic activities. Triterpene acids, including corosolic acid, oleanolic acid, and asiatic acid, are the principal phytochemicals in Banaba and are potentially anti-diabetic substances, owing to their effect on blood glucose concentration. Bioconversion of Banaba leaf extract (BLE) by *Lactobacillus plantarum* CBT-LP3 improved the glucose uptake, insulin secretion, and fat browning of this functional food. Furthermore, we identified asiatic acid, which was found to be increased by 3.8-fold during the *L. plantarum* CBT-LP3-mediated bioconversion process using metabolite profiling. Most previous studies have focused on corosolic acid, another triterpene acid that is a known anti-diabetic compound and is used to standardize BLE preparations. However, asiatic acid is the second most common of the triterpene acids and is also well known to have anti-diabetic properties. The present study has provided strong evidence that asiatic acid represents an alternative to corosolic acid as the most important active compound. These results suggest that the probiotic-mediated bioconversion of BLE may improve the anti-diabetic effects of this functional food. This implies that the consumption of a probiotic should be encouraged for people undergoing BLE treatment to improve its anti-diabetic effects.

## 1. Introduction

The tropical plant *Lagerstroemia speciosa* Pers. (Banaba), which is native to several parts of southeast Asia, has been used as a folk medicine for the treatment of diabetes and kidney diseases [1]. Recent studies have shown that *Lagerstroemia speciosa* has antioxidant [2,3], anti-inflammatory [3], anticancer [4], anti-obesity [5], and anti-diabetic [2,6] activities. Ongoing studies are aiming to identify the active components of Banaba leaf extract (BLE) that are responsible for its bioactivities. Moreover, a 28-day acute toxicity study, involving the oral administration of a single dose of BLE of 2000 mg/kg to rats, showed no major toxic effects [7]. Thus, BLE is a safe, natural bioactive plant material.

In the 21st century, type 2 diabetes (T2D) is recognized as a major public health problem and has a considerable impact on health expenditure [8]. In addition, diabetes directly affects the quality of life of patients and results in significant premature mortality [9]. Its high prevalence is associated with the consumption of unhealthy diets and insufficient exercise, resulting in high body mass index (BMI) and high fasting plasma glucose concentration in individuals of a broad range of ages [10]. Numerous studies have shown that individuals with a high BMI are much more likely to develop T2D [11].

The consumption of functional foods is considered to be an appropriate preventive strategy for diabetes in high-risk individuals. These supplements may be used alongside other substances to improve their therapeutic effects. A number of these supplementary foods have been shown to contain bioactive components that have beneficial effects for the prevention and management of T2D and obesity [12].

In the present study, we aimed to determine whether the anti-obesity and anti-diabetic effects of BLE could be improved using a bacterial conversion progress. Many previous studies have shown that microbially-mediated bioconversion (biotransformation) and fermentation can increase the bioactivities of various natural metabolites, which is of great interest in the food industry, health supplement, and pharmaceutical discovery fields [13,14]. For examples, *L. reuteri* and *Enterococcus faecalis* have a high bioconversion potential which is related the excellent radical scavenging activity [15]. Ginsenosides (Rb1, Rb2, and Rc) of ginseng are transformed to ginsenoside C-K by the human gut microbiome, which is well known to have anticancer effects in tumor cells [16]. Additionally, *Lactobacillus plantarum*-mediated bioconversion of red ginseng could efficiently generate compound K using ginsenoside Rd, resulted in increasing antioxidant properties [17]. *Pediococcus acidilactici*-derived bioconversion of *Magnolia* (M.) *denudata* extract could also promote increases in its bioactivities including antioxidative and anticancer activities [18].

Such microbial bioconversion is a cost-effective and eco-friendly means of generating therapeutic substances [19]. Among the useful microbes, probiotics have great potential as bioconverters in the food industry, because they can generate novel bioactive metabolites that are safe for inclusion in the diet [20].

*Lactobacillus* sp. are probiotics that have a long history of safe use in humans, have been classified as “Generally Recognized as Safe” by the Food and Drug Administration (FDA), and have received a “Qualified Presumption of Safety” by the EFSA [21]. Of these, *Lactobacillus planetarium* is well known as the mediator of kimchi fermentation, which produces compounds such as organic acids, short chain fatty acids, vitamins, and peptides [21,22,23]. Furthermore, *L. plantarum* fermentation improves the flavor of the products [24,25], which is another important contribution for the development of functional foods.

Therefore, we investigated whether the benefits of BLE could be improved using a probiotic-derived bioconversion (PMB) method, because this has previously been shown to increase the bioactivities of natural substances derived from medicinal plants [26]. Probiotics have been shown to generate bioactive substances, such as antimicrobials, growth-promoters, immune stimulators, and anti-inflammatory substances, through this bioconversion process [27,28], and thereby can improve the beneficial effects of phytochemicals [29].

## 2. Materials and Methods

### 2.1. Bacterial Strains and Culture

*L. plantarum* CBT-LP3 KCTC10782BP, which was isolated from kimchi, was obtained from the culture collection maintained at Cell Biotech Co., Ltd. (Gimpo, Republic of Korea). The cells were cultured for 18–24 h at 37 °C in MRS broth (Difco, Detroit, MI, USA) or M9 broth (Difco).

### 2.2. L. plantarum CBT-LP3 Mediated Bioconversion of BLE

The BLE subjected to *L. plantarum* CBT-LP3-mediated conversion was purchased from Daelim Product Co., Ltd. (Seoul, Republic of Korea). The BLE was obtained by ethanol extraction by percolation at high temperature, followed by concentration in a vacuum evaporator, and possibly further drying under vacuum. Maltodextrin was used as the carrier during drying. The BLE was completely dissolved in M9 broth for *L. plantarum* CBT-LP3 mediated conversion.

The conversion method was performed as follows. First, *L. plantarum* CBT-LP3 cells were cultivated in MRS at 37 °C overnight, then 3 × 10^4^ cfu were seeded into M9 broth containing BLE (50 mg/mL) and incubated at 37 °C for 72 h. Subsequently, the culture was centrifuged (12,000 rpm, 4 °C, 20 min) and the supernatant was collected and filtered (0.22 μm) prior to use in experiments.

### 2.3. Cell Lines

The 3T3-L1 preadipocyte and INS-1 rat insulinoma cell lines were obtained from the American Type Culture Collection (Manassas, VA, USA).

### 2.4. Determination of Cell Viability

The viabilities of the 3T3-L1 and INS-1 cells were determined using the MTT assay (Cell Counting Kit-8; Dojindo Laboratories, Tokyo, Japan). Each of the cell lines was seeded into 96-well plates (density 1 × 10^3^ cells per well) and, after treatment with each substance, the absorbance of each well was measured using a multifunctional microplate reader (SpectraMax M5; Molecular Devices, San Jose, CA, USA). Cell viability was expressed as a percentage of that of the control cells [30].

### 2.5. Differentiation of 3T3 L1 Cells

Preadipocytes were induced to differentiate into adipocytes after 48 h of culture in 96-well plates, when they had reached confluence. The cells were incubated in differentiation medium (10% FBS-DMEM containing dexamethasone, IBMX, and insulin) for 48 h, and then the culture medium was changed to one containing insulin alone and the cells were cultured for a further 72 h. Finally, the differentiated cells were matured by culture in 10% FBS-DMEM for 72 h. The extent of differentiation was assessed using the morphological changes and the accumulation of lipid droplets in the cells. After 8–9 days of culture, the differentiated cells were subjected to Oil Red O staining [31].

### 2.6. Glucose Uptake Assay in 3T3-L1 Adipocytes

The effect of each test substance on glucose uptake by the 3T3-L1 adipocytes was assessed using a previously described method [32]. Briefly, the cells in 96-well plates were incubated in glucose-free DMEM containing 10% FBS for 12 h, then washed and incubated with each substance in 1 mM 2-deoxyglucose-containing reaction buffer for 10 min. Insulin was used as a comparator. Glucose uptake was assayed using a Glucose Uptake-Glo^TM^ Assay kit (Promega, Madison, WI, USA).

### 2.7. Oil Red O Staining

Differentiated 3T3-L1 cells were washed with DPBS, fixed in formalin, permeabilized using 0.5% Triton-X 100, and stained with Oil Red O at a final concentration of 0.14 mM. The stain was then eluted from the cells using 100% isopropanol and the absorbance of the eluted stain was measured at 490 nm. Photomicrographs of the cells were obtained using a Nikon Digital Imaging System (E200 HD), as previously described [33].

### 2.8. Analysis of the Expression of Signaling-Related Genes Using qPCR

RNA was extracted from cells using an RNeasy Mini Kit (Qiagen, Hilden, Germany), then reverse transcribed to complementary DNA (cDNA) using TOPscriptRT DryMIX dN6plus (Enzynomics, Daejeon, Republic of Korea) and the following thermocycling parameters: 25 °C for 10 min, 42 °C for 60 min, and 95 °C for 5 min. TB Green Premix Ex Taq II (Takara, Shiga, Japan) and a CFX Opus 96 real-time PCR system (Bio-Rad, Hercules, CA, USA) were used for qPCR. The thermocycling conditions were as follows: initial denaturation at 95 °C for 5 min; 40 cycles of 95 °C for 15 s, 60 °C for 30 s, and 72 °C for 30 s; and a final elongation at 72 °C for 10 min. The reference gene was *Actb*. Target gene expression was quantified using the comparative 2^−ΔΔCq^ method [34]. The primer sequences are listed in Supplementary Table S1.

### 2.9. Insulin Secretion Assay

INS-1 cells were seeded at an initial density of 3 × 10^5^ cells/well in 96-well plates. After incubation for 24 h, they were stimulated with glucose (5.5 mM and 33 mM in Krebs buffer (24 mM NaHCO_3_, 1.2 mM MgCl_2_, 1 mM HEPES, 129 mM NaCl, 4.8 mM KCl, 1.2 mM KH_2_PO_4_, 2.5 mM CaCl_2_, pH 7.4)) in the presence of 2% of test substance. Supernatants were transferred to 96-well plates and the insulin concentrations in each well were measured using a Human Insulin ELISA kit (Abcam, Cambridge, UK). Absorbances were measured using a multifunctional microplate reader (SpectraMax M5; Molecular Devices). The results quoted were obtained from three independent experiments.

### 2.10. Reactive Oxygen Species (ROS) Scavenging Assay

ROS scavenging was assessed using a live-cell imaging method and CellROX Deep Red reagent [35]. INS-1 cells (1 × 10^4^ cells/well) were seeded into 96-well plates, then CellROX reagent was added to a final concentration of 5 μM in 10% FBS-RPMI 1640 medium, and the plates were incubated for 30 min at 37 °C. Fluorescence images were acquired using GloMax Explorer (Promega), with excitation at 627 nm and emission at 660–720 nm.

### 2.11. Metabolomic Profiling

#### 2.11.1. Metabolomic Analysis

Metabolomic profiling was performed using a Q Exactive™ Hybrid Quadrupole-Orbitrap MS (Thermo Fisher Scientific, Waltham, MA, USA) coupled to a 1290 Infinity UHPLC (Agilent, Santa Clara, CA, USA). Metabolite mixtures were separated using a Zorbax Eclipse Plus C18 Rapid Resolution High Definition column (2.1 × 50 mm, 1.8 μm particles) with solvent A (water with 0.1% formic acid) at a flow rate of 0.2 mL/min. The metabolites were separated by passing 2.5% solvent B (80% acetonitrile with 0.1% formic acid) for 2 min, 2.5–12% solvent B for 11 min, 12–28% solvent B for 15 min, 28–60% solvent B for 22 min, 60–96% solvent B for 22 min, 96% solvent B for 26 min, and 2.5% solvent B for 26 min. Ionization was performed using a Heated Electrospray Ionization (HESI-II) Probe in combination with a standard Thermo Scientific™ Ion Max source [36].

#### 2.11.2. Metabolomic Data Analysis

The UHPLC-Orbitrap-MS/MS RAW files obtained were processed using Compound Discoverer™ 3.2.0.421 (Thermo Fisher Scientific). A non-targeted metabolomic workflow was used to perform retention time alignment and compound identification. Compounds were identified using mzCloud and ChemSpider [37]. Principal component analysis and heatmap construction were performed using MetaboAnalyst (http://www.metaboanalyst.ca/MetaboAnalyst, accessed on 26 August 2021, ver. 5.0) [38].

The Volcano plot was generated using MetaboAnalyst 5.0 to compare the metabolite changes between the BBLE and BLE groups. The entire data processing was followed each step. First step, the raw files obtained through LC-MS analysis were searched for metabolites using Compound Discoverer 3.3. and then, to improve the quality of the data, the search results were filtered by Levels 2 and 3, and solvent compounds and unannotated peaks were removed [39]. The criteria for Levels 2 and 3 were defined by the Metabolomics Society. As second step, the final data was obtained after removing duplicate results and then statistical analysis was performed in MetaboAnalyst 5.0. The data were normalized by the sum method and log10 transformed and autoscaled. Sum-based normalization is a widely used method in metabolite data analysis which is used to adjust data values based on the sum of all abundance values in each sample. Through this normalization and transformation process, the data distribution was standardized and the reliability of the analysis was increased. As a last step, Student’s *t*-test was performed to analyze differentially expressed metabolites (DEMs) between the two groups based on the normalized data resulting in a volcano plot being created. The x-axis shows the log2-transformed fold change, and the y-axis shows the −log10 *p*-value. The color-shaded plot targets DEMs with an FC absolute value of 2 or more and a *p*-value standard of 0.05 or less, and the *p*-value threshold was set to control false positives.

### 2.12. Chromatography for the Quantification of the Ursolic Acid and Asiatic Acid Concentrations

An Eclipse Plus C18 chromatography column (4.6 mm × 250 mm, 5 μm; Waters, Milford, MA, USA) was used. The mobile phases were HPLC-grade water (solvent A) and acetonitrile (solvent B). Gradient elution was used to elute asiatic acid and ursolic acid from the column (0–20 min, 40% A, 60% B→0% A, 100% B; 20–25 min, 0% A, 100% B; 25–27 min, 0% A, 100% B→40% A, 60% B; 27–30 min, 40% A, 60% B). The flow rate was set at 1.0 mL/min and the temperature was maintained at 35 °C. Detection was performed at a wavelength of 205 nm. The injection volume was 10 μL.

### 2.13. Statistical Analysis

All data of statistical analysis were statistically analyzed using the GraphPad Prism^®^8 (Graphpad Software Inc., Boston, MA, USA) program. Each experiment was performed more than three times independently unless stated otherwise and data are presented as the mean ± standard deviation. In order to verify the significance between each experimental group, one-way ANOVA and Tukey’s post hoc test were used for analysis. *p* < 0.05 was considered to indicate statistical significance.

## 3. Results

### 3.1. Effect of BLE with or without L. plantarum CBT-LP3-Mediated Bioconversion on the Viability of Each Cell Line

**Figure 1:**
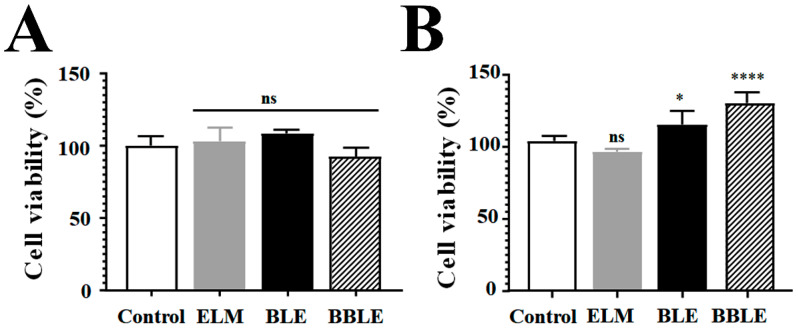
Cytotoxicity of each substance. The cytotoxicity of each test substance was assessed prior to further experiments. (**A**) Two-microliter (2% *v*/*v*) aliquots of each substance were added to 3T3-L1 cells for 48 h, and the viability of the cells was then determined using a multiplate reader and a WST-8 Cell Viability Assay Kit. The data are the mean ± SEM of three independent experiments (*n* = 6). (**B**) Two-microliter (2% *v*/*v*) aliquots of each substance were incubated with INS-1 cells for 48 h, and the cell viability was then determined using a microplate reader and a WST-8 Cell Viability Assay Kit. The data are the mean ± SEM of three independent experiments (*n* = 5). * *p* < 0.05 and **** *p* < 0.0001 vs. control.

3T3L-1 cells were used to assess the effects of the test substances on glucose uptake and adipocyte differentiation, and INS-1 cells were used to assess their effects on insulin secretion and the recovery from streptozotocin (STZ)-induced damage. Initially, the in vitro toxicity of BLE derivatives was tested by evaluating the viability of each cell line following treatment (Figure 1). The extent of inhibition by each substance did not exceed the IC_80_, implying that the use of BLE derivatives at concentrations below 2% (1 mg/mL) is safe.

### 3.2. Effect of L. plantarum CBT-LP3-Bioconverted Banaba Leaf Extract on Glucose Uptake

**Figure 2:**
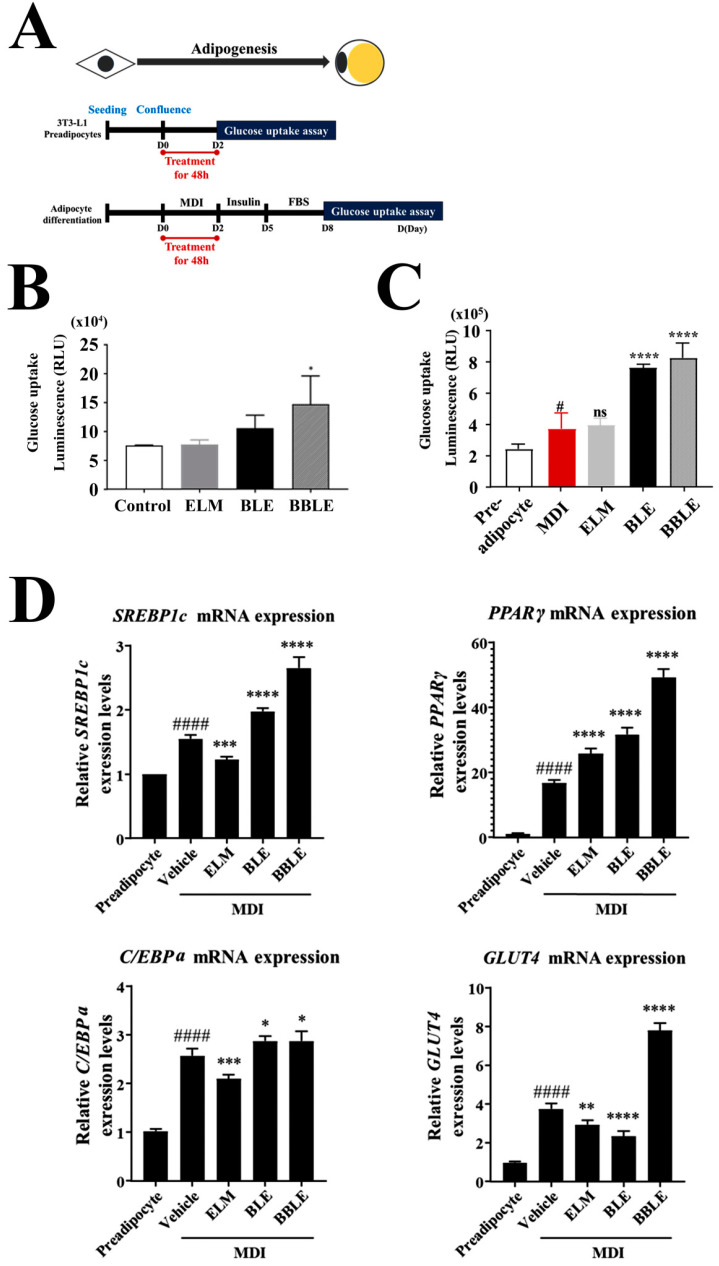
Effects of each substance on glucose uptake by 3T3-L1 cells. (**A**) Scheme for the experiment. (**B**) Glucose uptake, determined at the pre-adipocyte stage, in the absence of methylxanthine, dexamethasone, and insulin (MDI) treatment. The data are the mean ± SEM of three independent experiments (*n* = 3). * *p* < 0.05 vs. control. (**C**) Glucose uptake, determined after adipocyte differentiation resulting from the MDI treatment of 3T3-L1 cells, determined using a Glucose Uptake-Glo^TM^ Assay kit. The data are the mean ± SEM for three independent experiments (*n* = 4). # *p* < 0.05 vs. preadipocyte, **** *p* < 0.0001 vs. MDI. (**D**) Relative mRNA expression of glucose uptake-related genes in 3T3-L1 cells, measured using qPCR, with *Actb* as the reference gene. The data are the mean ± SEM of three independent experiments (*n* = 4). #### *p* < 0.0001 vs. preadipocyte, and ** *p* < 0.01, *** *p* < 0.001 and **** *p* < 0.0001 vs. vehicle.

Many previous studies have shown that BLE stimulates glucose uptake [40]. In addition, *L. plantarum* CBT-LP3 is known to increase the bioactivities of various natural substances by bioconversion and fermentation [41]. Therefore, we compared the effects of BLE and bioconverted Banaba leaf extract (BBLE) on glucose uptake by preadipocytes and mature adipocytes (Figure 2A). Strong stimulatory effects of both BLE and BBLE were obtained in both types of 3T3-L1 cells, compared with untreated cells (Figure 2B,C). At the preadipocyte stage, both BLE and BBLE caused slight increases, and BBLE had a more marked effect (Figure 2B). However, there were marked effects of both substances on the glucose uptake of the mature adipocytes (Figure 2C).

To investigate the molecular mechanism of these effects, we isolated mRNA from cells treated under each condition and measured the expression levels of genes encoding proteins involved in glucose uptake by qPCR (Figure 2D). *L. plantarum* CBT-LP3-mediated bioconversion (BBLE) resulted in an upregulation of genes encoding proteins involved in glucose uptake and the associated signaling pathway from SREBP1c to GLUT4, although BLE did not increase GLUT4 expression any more than differentiation alone (Figure 2D). Moreover, the expression of GLUT4 by BBLE, an effector of the insulin signaling pathway, was highly consistent with the results obtained for glucose uptake (Figure 2C). Thus *L. plantarum* CBT-LP3-mediated bioconversion might positively affect the expression of genes encoding proteins involved in glucose uptake resulting in a lowering of the blood glucose level.

### 3.3. Effect of L. plantarum CBT-LP3-Mediated Bioconversion on the Adipocyte Browning Stimulated by BLE

**Figure 3:**
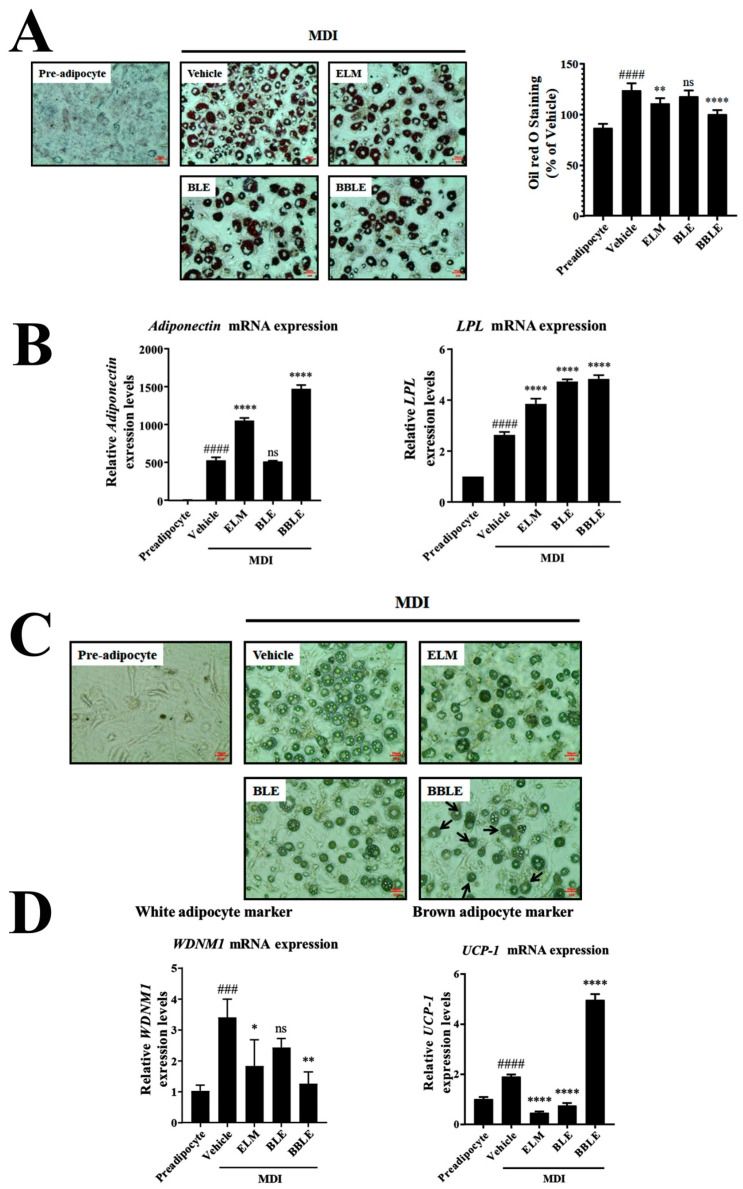
Induction of browning of 3T3-L1 cells by each substance. (**A**) To determine whether each substance affected the total lipid content of the 3T3-L1 cells, we examined the cells after ORO staining using a bright-field microscope (magnification ×4). The stain was then eluted from the cells using 100% isopropanol and the absorbance of the eluted stain was measured at 490 nm. The data are the mean ± SEM of three independent experiments (*n* = 5). #### *p* < 0.0001 vs. preadipocyte and ** *p* < 0.01 vs. vehicle. (**B**) To determine whether each substance affected the mRNA expression of lipolysis-related genes, their expression was measured using qPCR and *Actb* as the reference gene. The data are the mean ± SEM of three independent experiments (*n* = 4). #### *p* < 0.0001 vs. preadipocyte, **** *p* < 0.0001 vs. vehicle. (**C**) To determine whether each substance stimulated browning in 3T3-L1 cells, the number and size of the cells were counted using a bright-field microscope (magnification ×10). A low value indicated morphologically changed 3T3-L1 cells which are significantly reduced adipocytes in cytosol. (**D**) To determine whether each substance affected the mRNA expression of the white adipocyte marker gene (WDNM1) and brown adipocyte marker gene (UCP-1), their expression was measured using qPCR with *Actb* as the reference gene. The data are the mean ± SEM of three independent experiments (Left: *n* = 3, Right: *n* = 4). #### *p* < 0.0001 and ### *p* < 0.001 vs. preadipocyte, **** *p* < 0.0001, ** *p* < 0.01, and * *p* < 0.05, vs. vehicle.

A chronic imbalance in energy intake and expenditure leads to adipocyte hypertrophy and hyperplasia and thus obesity [42]. BBLE treatment not only increased glucose uptake by adipocytes (Figure 2C), but also increased the expression of relevant signaling intermediates (Figure 2D). Therefore, excess fat accumulation may result from the continuous stimulation of glucose uptake into adipocytes by BBLE. Therefore, we investigated whether BBLE might also trigger the differentiation of preadipocytes into brown adipocytes, which are thermogenic, and thereby prevent obesity. We first assessed the accumulation of fat droplets in the 3T3-L1 cells (Figure 3A,B), which may represent the effect of BBLE on lipolysis (Figure 3C,D).

To investigate the effects of each substance on lipogenesis, we also assessed fat droplet accumulation through Oil Red O (ORO) staining (Figure 3A) and investigated effects on the expression of genes encoding proteins (adiponectin and lipoprotein lipase (LPL)) involved in lipolysis (Figure 3B). When normal mature adipocytes were used as the control, BBLE treatment showed the poor fat droplets in the cells after ORO staining (Figure 3A). Furthermore, gene expression levels of adiponectin and LPL strongly supported reduction in lipid storage droplets in adipocytes. These results suggested that BBLE treatment reduced lipid accumulation in the cells more than mature adipocytes (vehicle) (Figure 3A,B), despite BBLE significantly increasing glucose uptake by the mature adipocytes (Figure 2C,D).

We further investigated the anti-lipogenic effects of BBLE in 3T3-L1 cells by examining the lipolysis morphology of the fat droplets (Figure 3C) and measuring the expression of UCP1 and WDNM1 by qPCR (Figure 3D). The UCP1 gene was used as a white adipocyte marker and the WDNM1 gene was used as a brown adipocyte marker, respectively. As shown in Figure 3C, BBLE significantly increased the lipolysis phenomena resulting in a reduction in the number of lipid droplets and their size. Additionally, we found the lipolysis phenomena was related with adipocyte browning, because BBLE specifically increased UCP1 expression level, whereas it significantly reduced WDNM1 (Figure 3D). BLE had slightly positive effects on glucose uptake and lipolysis adipocytes, but a lesser effect on the gene expression mediating glucose uptake and fat browning. Therefore, *L. plantarum* CBT-LP3-mediated bioconversion increases the anti-obesity effects of BLE.

### 3.4. Effects of L. plantarum CBT-LP3-Mediated Bioconversion on the Stimulation of Insulin Secretion and STZ Recovery by Banaba Leaf Extract

**Figure 4:**
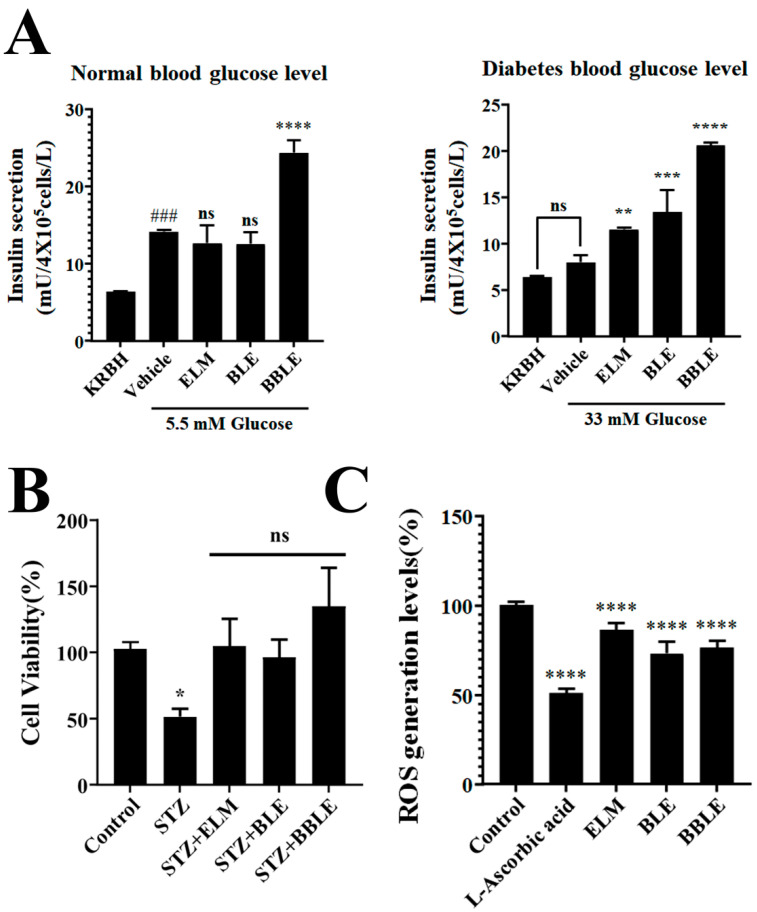
Effect of each substance on insulin secretion. (**A**) To determine the effects of each substance on insulin secretion by INS-1 cells, the cells were incubated under normal and high-glucose conditions, and the insulin concentration of the medium was measured using a Human Insulin ELISA kit. The data are the mean ± SEM of three independent experiments (Left: *n* = 3, Right: *n* = 3). ### *p* < 0.001 vs. KRBH, **** *p* < 0.0001, *** *p* < 0.001, ** *p* < 0.01 vs. vehicle. (**B**) To evaluate the recovery of INS-1 cells from STZ-induced damage in the presence of each test substance, a WST-8 Cell Viability Assay Kit was used. The data are the mean ± SEM of three independent experiments (*n* = 3). * *p* < 0.01 vs. control. (**C**) To determine how each substance could affect cell survival following TBHP treatment, which rapidly increases ROS production by cells, the ROS-scavenging properties of each substance in INS-1 cells was investigated using L-ascorbic acid as a positive control. The data are the mean ± SEM of three independent experiments (*n* = 6). **** *p* < 0.0001 vs. control.

Defective insulin secretion by pancreatic beta cells is a key pathophysiological defect in type 2 diabetes mellitus (T2D) [43]. Therefore, the stimulation of insulin secretion by beta cells should be beneficial for patients with T2D. As a consequence, we assessed insulin secretion by rat insulinoma INS-1 cells and determined whether BBLE increases insulin secretion more than BLE (Figure 4A).

Initially, we assessed the effect of glucose concentration on insulin secretion by INS-1 cells (normal-glucose: 5.5 mM and high-glucose: 33 mM). Normal glucose conditions provoked a 3.5-fold increase in insulin secretion by the cells, whereas high-glucose conditions reduced this to a 2-fold increase. BBLE significantly increased insulin secretion by 4.5–6-fold under all these conditions. In contrast, BLE or an extract of *L. plantarum* CBT-LP3 media (ELM) alone had no effect on insulin secretion under low-glucose and normal glucose conditions, and only caused slight increases under high-glucose conditions. Thus, *L. plantarum* CBT-LP3-mediated bioconversion of BLE has a potent effect of increasing insulin secretion by cultured pancreatic beta cells.

Insulin secretion may be reduced by the loss of or damage to pancreatic beta cells. Therefore, we investigated whether BBLE can improve the recovery of STZ-damaged INS-1 cells. STZ is well known to be toxic for beta cells, but although STZ (12.5 mM) treatment reduced the viability of the cells to 37% of control levels, both ELM and BLE caused a recovery of viability up to control levels (Figure 4B). Interestingly, BBLE caused a recovery up to 134% of control levels.

STZ is also well known to induce the apoptosis of INS-1 cells, which is mediated through an increase in ROS concentration [44]. Therefore, we investigated whether BLE or BBLE scavenge ROS in INS-1 cells. We treated cells with tert-butyl hydroperoxide (TBHP) 1.2 mM to induce ROS production, without causing severe toxicity (Figure 4C). We confirmed that this was not toxic alone or in combination with each of the test substances, alongside L-ascorbic acid as a positive control. We assessed ROS scavenging by each substance by calculating the reduction in fluorescence intensity and found that BLE and BBLE had similar ROS-scavenging effects (Figure 4C). Thus, the substantial effect of BBLE on STZ recovery may be explained by the greater cell proliferative effect of BBLE (Figure 1B) than BLE.

### 3.5. Metabolomic Profiling of L. plantarum CBT-LP3-BBLE

**Figure 5:**
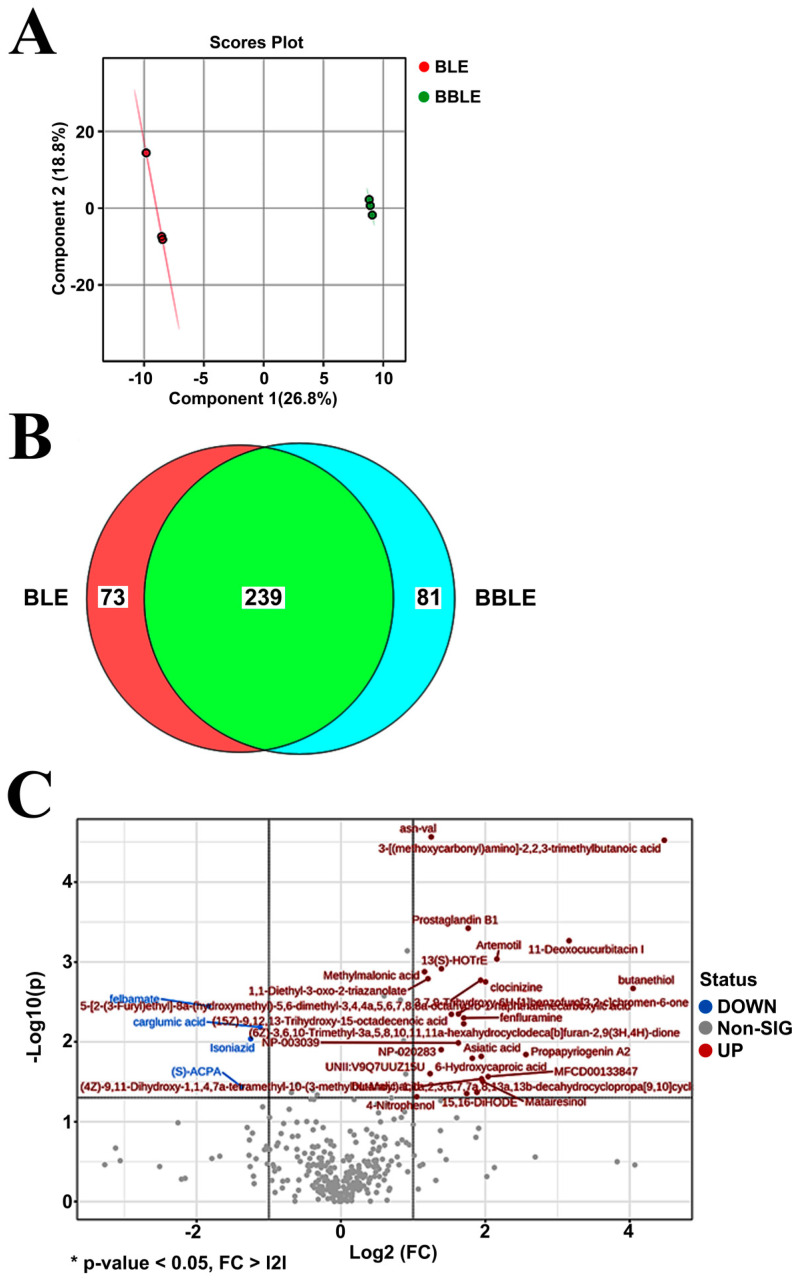
Comparison of the metabolomic profiles of BLE and BBLE. A PLS-DA score plot and Venn diagram derived from the LC-MS data are shown. (**A**) Partial least-squares discriminant analysis (PLS-DA) was performed to obtain a global overview of the difference in the metabolites present in BLE and BBLE. (**B**) Venn diagram of the number of annotated metabolites in BLE and BBLE, obtained using negative ion mode. (**C**) Volcano plot of the metabolites present at differing concentrations in BLE and BBLE. The *p*-value was calculated using Student’s *t*-test. Red dots denote significant (*p* < 0.05) differences with fold changes >2; blue dots represent significant differences (*p* < 0.05), black dots represent a fold change >2, and clear dots represent no significance (*p* > 0.05) and a fold change < 2.

We used metabolomic profiling to quantify differences in the contents of active compounds in the samples [45] that might have mediated the superior anti-diabetic activities of BBLE (Figure 2, Figure 3 and Figure 4). Partial least-squares discrimination analysis (PLS-DA) was performed to obtain a global overview of the differences in metabolite concentrations in BLE and BBLE, and score and loading plots are shown in Figure 5A. Three BLE and BBLE samples were evaluated and found to be clustered into two groups on the basis of scores based on negative ion mode data. PLS 1 and 2 described 45.6% of the total variation between the metabolomic profiles of the two types of sample. Thus, there were significant differences in the composition of BLE and BBLE.

A total of 312 and 320 metabolites were identified in BLE and BBLE, respectively, in negative mode (Supplementary Table S2), and the two types of sample shared 239 metabolites (Figure 5B). Eighty-one metabolites that may have been converted or synthesized during the *L. plantarum* CBT-LP3-mediated bioconversion process were identified in BBLE. In the negatively ionized mode, 393 annotated metabolites were identified, of which 27 were present in higher concentrations and 4 were present in lower concentrations in BBLE than in BLE (fold change > 2, *p* < 0.05) (Table 1 and Figure 5C). Asiatic acid was one of the 27 metabolites that were present at higher concentrations, the concentration of which was increased by 3.8-fold by *L. plantarum* CBT-LP3-mediated bioconversion (Table 1). Furthermore, we also confirmed that ursolic acid was converted to asiatic acid during the incubation of *L. plantarum* CBT-LP3 in M9 media containing ursolic acid (50 mg/mL) at 37 °C for 72 h.

**Figure 6:**
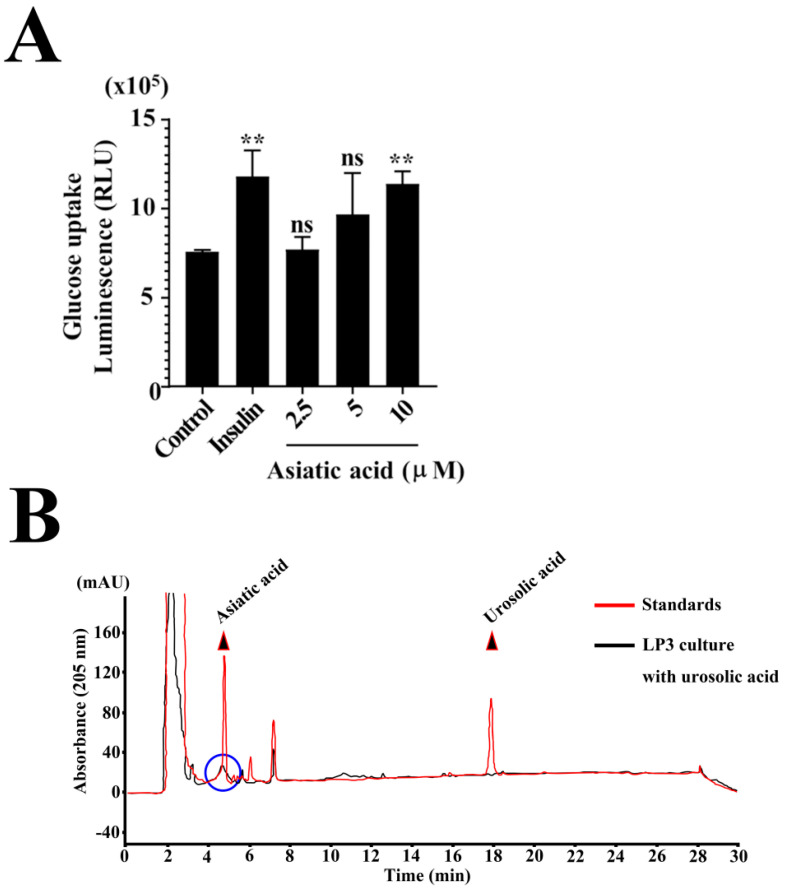
*L. plantarum* CBT-LP3-mediated conversion of ursolic acid to asiatic acid. (**A**) To determine whether asiatic acid can stimulate glucose uptake in 3T3-L1 cells, we measured the glucose uptake in the presence of insulin (0.25 nM) and asiatic acid in 3T3-L1 cells at the preadipocyte stage. The data are the mean ± SEM of three independent experiments (*n* = 4). ** *p* < 0.01 vs. control. (**B**) To evaluate the *L. plantarum* CBT-LP3-mediated bioconversion of ursolic acid to asiatic acid, *L. plantarum* CBT-LP3 cells (10^4^ cfu) were incubated in M9 culture media in the presence of ursolic acid (100 μg/mL) at 37 °C for 72 h. The two polar metabolites, ursolic acid and its product asiatic acid (blue circle), were extracted using a double volume of butanol (*v*/*v*), which was then completely evaporated. The mixture was resuspended in methanol and then HPLC analysis was performed.

Many previous studies have demonstrated that asiatic acid is one of the main phytochemical components of BLE [46,47], and it has been shown to inhibit glucose uptake [48]. We also confirmed that asiatic acid showed stimulation of glucose uptake depending on the concentration (Figure 6A). To further evaluate the *L. plantarum* CBT-LP3-mediated bioconversion of ursolic acid to asiatic acid, *L. plantarum* CBT-LP3 cells (10^4^ cfu) were cultured in M9 culture media containing ursolic acid (100 μg/mL) at 37 °C for 72 h which resulted in all of the ursolic acid being converted to asiatic acid (Figure 6B). This finding is consistent with the increase in the asiatic acid content induced by *L. plantarum* CBT-LP3-mediated bioconversion being responsible for the greater glucose uptake associated with the BBLE treatment of 3T3-L1 cells (Figure 6).

## 4. Discussion

The rising prevalence of obesity and T2D has led to considerable attention being devoted to the discovery of novel therapeutics and health supplements, alone or in combination [49]. In the present study, we evaluated the effects of the probiotic-mediated conversion of BLE, an example of a medicinal herb that is claimed to have several beneficial effect in patients with T2D [50,51]. Many previous studies have demonstrated that lactic acid-producing bacteria or probiotics produce beneficial metabolites from herb extracts [51].

We performed bio-conversion using *L. plantarum* CBT-LP3 which has the properties of a good starter strain [52,53]. Superior effects of this product were achieved with respect to all the anti-diabetic indices assessed. We first compared the effects of BLE and BBLE on glucose uptake by 3T3-L1 cells in their preadipocyte and mature adipocyte forms. We found that *L. plantarum* CBT-LP3-mediated bioconversion caused a two-fold increase in uptake vs. BLE in both cell types (Figure 2B,C), along with consistent increases in the expression of related genes (Figure 2D). In addition, we found that the components of the SREBP1-PPARγ-GLUT4 pathway in 3T3-L1 cells were strongly upregulated by BBLE, whereas that of C/EBPα was not (Figure 2D).

We also evaluated the brown adipocyte characteristics of the cells. Mammals have two types of adipose tissue: white and brown. White adipose tissue (WAT) contains adipocytes with a single large fat droplet that is used for energy storage, and adipocytes differentiate from undifferentiated preadipocytes in response to the expression of key transcription factors, such as PPARγ, which is required for both adipogenesis and the maintenance of adipocytes. By contrast, brown adipose tissue (BAT) helps to maintain body temperature through non-shivering thermogenesis. BAT releases energy in the form of heat as a result of the uncoupling of the respiratory chain from ADP phosphorylation through the effects of uncoupling protein 1 (UCP1). Because of this, an increase in the number of brown adipocytes or BAT-like activity in WAT prevents diet-induced obesity and reduces the incidence and severity of T2D. Thus, the conversion of fat-storing cells in WAT to metabolically active thermogenic cells, as present in BAT, has become an appealing therapeutic strategy to combat obesity [54].

To further evaluate the potential browning activity of BLE or BBLE, we evaluated their effects on the lipid droplet morphology of the fully differentiated 3T3-L1 cells (Figure 3A). BLE and BBLE were found to induce brown-like lipid morphology (Figure 3C, arrows), and BBLE significantly reduced lipid droplet size and number in the 3T3-L1 cells. These results suggest that BBLE not only significantly increases glucose uptake (Figure 2C,D), but also causes the browning of 3T3-L1 cells (Figure 3C,D). Furthermore, we demonstrated higher expression of brown fat-specific genes in response to BBLE treatment, including those of UCP1 and adiponectin, and a lower expression of the WAT-specific gene WDNM1 (Figure 2C and Figure 3C). These results suggest that BBLE may aid the control of blood glucose in patients with T2D and prevent obesity by inducing thermogenesis.

We also found that BBLE stimulates insulin secretion in the presence of a broad range of glucose concentrations (Figure 4A), that it stimulates growth under normal conditions (Figure 1B), and that it improves cellular recovery following STZ treatment (Figure 4B) using INS-1 cells. BLE and BBLE were also shown to have a ROS-scavenging effect, which might explain the reduction in the STZ-induced apoptosis of INS-1 cells (Figure 4C).

We attempted to identify candidate anti-diabetic substances in the Banaba leaf extracts. Previous metabolite profiling of 17 different Banaba species identified four triterpene acids (asiatic acid, corosolic acid, virgatic acid, and oleanolic acid/ursolic acid) as such candidates [48], and when we compared the metabolomic profiles of BLE and BBLE, asiatic acid was found to be 1 of 31 candidates that were present at two-fold higher or lower concentrations in the latter. Specifically, the concentration of asiatic acid was increased 3.8-fold by the *L. plantarum* CBT-LP3-mediated conversion process (Figure 5C). According to Kim et al. (2020), BLE contains 1.956 mg/g of asiatic acid, which implies that BBLE should contain approximately 7.4 mg/g. This quantity is larger than that measured for corosolic acid (5.424 mg/g), which is one of the candidate anti-diabetic substances in BLE. Furthermore, Kim’s group demonstrated that asiatic acid has a larger effect on glucose uptake than corosolic acid in L6-GLUT4myc cells [48]. Figure 6A shows that asiatic acid has a dose-dependent effect on glucose uptake. Finally, we confirmed that *L. plantarum* CBT-LP3 converts ursolic acid to asiatic acid (Figure 6).

Furthermore, Artemotil is well known as the treatment for chloroquine-resistant Plasmodium falciparum malaria and cerebral malaria cases. It is a fast-acting blood schizonticide and currently only used as a second-line drug in severe cases of malaria [55]. Testosterone deficiency is associated with increased obesity risks such as increased fat storage, insulin resistance, and worsening glycemic control [56]. Therefore, testosterone replacement therapy has been used to treat hypogonadal males with T2DM for a long time, despite variable results [57]. Clocinizine is a first-generation antihistamine of the diphenylmethylpiperazine class and it has marketed in Spain in combination with phenylpropanolamine under the brand name Senioral [58]. Matairesinol, an organic compound, is classified as a lignan and it is present in some cereals, such as rye. Matairesinol has attracted much attention for its beneficial nutritional effects. Matairesinol has been found to act as an agonist of the adiponectin receptor 1 (AdipoR1) [59]. Fenfluramine has formerly been used as an appetite suppressant in the treatment of obesity, but it was withdrawn for this use due to cardiovascular toxicity [60].

In conclusion, Banaba leaves have been used as a folk medicine for the control of blood glucose in patients with diabetes, and aqueous and methanol extracts have been used as dietary (food, health, or nutritional) supplements. Most previous studies have focused on corosolic acid, a triterpene acid that has anti-diabetic effects, and has therefore been used to standardize BLE [61]. However, some studies, including the present study, show that asiatic acid may represent an alternative candidate anti-diabetic substance to corosolic acid [48,62]. The key findings of the present study are that the *L. plantarum* CBT-LP3-mediated conversion process significantly increases the anti-diabetic effects (on glucose uptake, insulin secretion, the recovery of pancreatic beta cells from damage, and ROS scavenging) of BLE, which may be mediated by the 3.8-fold increase in the asiatic acid content of BBLE.

Thus, this study represents a promising direction. The consumption of probiotics alongside BLE can be recommended to patients to improve the anti-diabetic effects of this medicinal plant. Furthermore, to understand how or why probiotics can use phytochemicals, we will need in-depth study about their metabolism and pathways.

## Figures and Tables

**Table 1:** Results of metabolomic profiling in negative mode.

Metabolite	FC	Up or Down Regulation
3-[(Methoxycarbonyl)amino]-2,2,3-trimethylbutanoic acid	22.31	UP
Butanethiol	16.528	UP
11-Deoxocucurbitacin I	8.9384	UP
Propapyriogenin A2	5.9063	UP
Artemotil	4.4698	UP
Testosterone decanoate	4.1097	UP
Clocinizine	4.0083	UP
Matairesinol	3.9018	UP
DL-Malic acid	3.8591	UP
Asiatic acid	3.8478	UP
5-[2-(3-Furyl)ethyl]-8a-(hydroxymethyl)-5,6-dimethyl-3,4,4a,5,6,7,8,8a-octahydro-1-naphthalenecarboxylic acid	3.8229	UP
(4Z)-9,11-Dihydroxy-1,1,4,7a-tetramethyl-10-(3-methylbutanoyl)-1,1a,2,3,6,7,7a,8,13a,13b-decahydrocyclopropa [9,10] cyclodeca [1,2-b]chromene-12-carbaldehyde	3.6902	UP
6-Hydroxycaproic acid	3.5304	UP
Prostaglandin B1	3.3927	UP
15,16-DiHODE	3.3456	UP
Fenfluramine	3.2537	UP
(6Z)-3,6,10-Trimethyl-3a,5,8,10,11,11a-hexahydrocyclodeca[b]furan-2,9(3H,4H)-dione	3.2519	UP
3,7,9-Trihydroxy-6H-[1] benzofuro [3,2-c]chromen-6-one	3.0903	UP
NP-003039	3.0869	UP
(15Z)-9,12,13-Trihydroxy-15-octadecenoic acid	2.8871	UP
13(S)-HOTrE	2.6243	UP
NP-020283	2.6179	UP
Asn-Val	2.3794	UP
UNII:V9Q7UUZ15U	2.354	UP
1,1-Diethyl-3-oxo-2-triazanolate	2.3058	UP
Methylmalonic acid	2.2318	UP
4-Nitrophenol	2.0699	UP
Carglumic acid	0.45971	DOWN
Isoniazid	0.42035	DOWN
(S)-ACPA	0.38543	DOWN
Felbamate	0.28071	DOWN

The target metabolite is indicated in red.

## Data Availability

The original contributions presented in the study are included in the article/Supplementary Material, further inquiries can be directed to the corresponding author.

## Supplementary Materials

The following supporting information can be downloaded at: https://www.mdpi.com/article/10.3390/microorganisms12091848/s1, Table S1: List of primers. Table S2: List of annotated metabolites in BLE and BBLE.
