# Supplementary material for: Identification of Bioactive Substances Derived from the Probiotic-Induced Bioconversion of Lagerstroemia speciosa Pers. Leaf Extract That Have Beneficial Effects on Diabetes and Obesity
Source: Microorganisms. 2024 Sep 6;12(9):1848. doi: 10.3390/microorganisms12091848 (PMC11434581; doi:10.3390/microorganisms12091848)
Supplement: Supplementary file 1 [file microorganisms-12-01848-s001.zip › Supplementary table S1.pdf]

Supplementary table S1. List of primers

Gene name	Primer sequences	
	Forward (5′→3′)	Reverse (5′→3′)
**PPARα**	AGG CTG TAA GGG CTT CTT TCG	GGC ATT TGT TCC GGT TCT TC
**PPARγ**	CGC TGA TGC ACT GCC TAT GA	AGA GGT CCA CAG AGC TGA TTC C
**SREBP1c**	TGTTGGCATCCTGCTATCTG	AGGGAAAGCTTTGGGGTCTA
**GLUT4**	CCAACAGCTCTCAGGCATCA	TGACCACACCAGCTCCTATGG
**C/EBPα**	AAA CAA CGC AAC GTG GAG A	GCG GTC ATT GTC ACT GGT C
**Adiponectin**	AGG GAG AGA AAG GAG ATG CAG	CTT TCC TGC CAG GGG TTC
**FGF21**	ACACAATTCCAGCTGCCTTG	TAGAGGCTTTGACACCCAGG
**WDNM1**	CCTGGGCTCTGTCTAACC	CATCGTTCATCACAAGTTCC
**UCP-1**	GATGGTGAACCCGACAACTT	CTGAAACTCCGGCTGAGAAG
**Actb**	GACTTCGAGC AAGAGATGGC	CCAGACAGCA CTGTGTTGGC
