# Supplementary material for: Identification of Bioactive Substances Derived from the Probiotic-Induced Bioconversion of Lagerstroemia speciosa Pers. Leaf Extract That Have Beneficial Effects on Diabetes and Obesity
Source: Microorganisms. 2024 Sep 6;12(9):1848. doi: 10.3390/microorganisms12091848 (PMC11434581; doi:10.3390/microorganisms12091848)
Supplement: Supplementary file 1 [file microorganisms-12-01848-s001.zip › Supplementary table S2.pdf]

Negative mode		
**Only Leaf (73)**	**Common (239)**	**Only LP3 (81)**
Paraxanthine	1,5-anhydroglucitol	3-Methoxy-19-norpregna-1,3,5(10)-trien-20-one
erythrose	Enprofylline	1-O-Phosphonopentitol
1-(((5-amino-2-furanyl)methylene)amino)-2,4-imidazolidinedione	Theobromine	D-Xylonolactone
Succinylacetone	Levonorgestrel	MFCD11045308
(+/-)-2-Hydroxyglutaric acid	Nicorandil	2-AMINO-4,6-DINITROTOLUENE
1-Deoxy-L-mannitol	4-Nitrophenol	(2S,4S)-4-Amino-2-hydroxy-2-methylpentanedioic acid
2-amino-2,3,7-trideoxy-D-lyxo-hept-6-ulosonic acid	Metronidazole	DO0750000
3,5-di-tert-Butyl-4-hydroxybenzyl alcohol	N-Acetyl-L-histidine	3-[(methoxycarbonyl)amino]-2,2,3-trimethylbutanoic acid
nihydrazone	D-(-)-Fructose	3-[(Z)-(Hydroxy{(2R,3R)-3-[hydroxy(imino)methyl]-2-oxiranyl}methylene)amino]-L-alanine
fenamole	L-Tyrosine methyl ester	2-isopropylmaleic acid
1,5-Anhydro-D-erythro-hexo-2,3-diulose	Acetone peroxide	.alpha.-Aminoadipic acid
Ethyl 2,4-dioxohexanoate	2,2-Methylenebis(4-ethyl-6-tert-butylphenol)	N-glycoloyl-beta-D-glucosamine
UP0783700	(+)-[6]-Gingerol	beta-D-Ethyl glucuronide
7-Cyano-7-deazaguanine	4-Methoxy-3,4a,5-trimethyl-4,4a,5,6,7,8,8a,9-octahydronaphtho[2,3-b]furan	cis-4-Octenedioic acid
N-Formyl-L-glutamic acid	2,3,4,5-Tetrahydroxypentanal	MFCD00056763
PKF118-310	Methylglutaric acid	1,1-Diethyl-3-oxo-2-triazanolate
6-(Hydroxymethyl)-2,4(1H,3H)-pteridinedione	1,5-Anhydro-D-fructose	4-Oxoproline
8-(3,4-Dihydroxyphenyl)-5-hydroxy-7-methoxy-6H-[1,3]dioxolo[4,5-h]chromen-6-one	Pyrophosphoric Acid	Suberic acid
(E)-Nitrofurazone	2-Deoxypentose	Aminoparathion
Carbendazim	Alchorneine	alpha-Ketoglutaric acid
2-amino-3-oxoadipic acid	Ellagic acid	1-Hydroxy-1-(4-methoxyphenyl)-4-methyl-3-pentanone
4-Hydroxy-2-oxoglutaric acid	isopropylmalic acid	Undecanedioic acid
(2R)-2,3-Dihydroxypropanoic acid	6-Deoxy-D-arabino-hexos-2-ulose	DL-Malic acid
CV5860000	Procarbazine	2-[(2E)-5-(3,3-Dimethyl-2-oxiranyl)-3-methyl-2-penten-1-yl]-1,3,5-benzenetriol
6-[(1R,2S)-1,2-Dihydroxypropyl]-3,4-dihydro-2,4-pteridinediol	threonic acid	deoxyuridine
RV0540000	S-(−)-Willardiine	Prostaglandin B1
nifuradene	Arabic acid	8-hydroxy-7-methylguanine
SL9650000	Xylitol	Arabinosylhypoxanthine
MFCD00041500	1,4,9-Trihydroxy-9-methyl-5,8,10-trioxo-5,8,9,10-tetrahydro-2-phenanthrenecarboxylic acid	MFCD20488630
Tocotrienol	Erythritol	1,7-Dihydroxy-6,6-dimethyl-3,5,5a,6,7,8,9a,9b-octahydronaphtho[1,2-c]furan-9(1H)-one
NP-018716	Succinic acid	Methyl thiobutyrate
Nicotinuric acid	6-Hydroxycaproic acid	ET5956200
3-tert-Butyladipic acid	3,4-Dihydroxyphenylglycol	(4R)-4-Hydroxy-5-oxo-L-norvaline
2-O-ETHYL ASCORBIC ACID	D-Glucono-delta-lactone	clocinizine
fenoxycarb	4-amino-2-hydroxyamino-6-nitrotoluene	14,18-Dihydroxy-12-oxo-9,13,15-octadecatrienoic acid
Dyphylline	Ethylmalonic acid	Corchorifatty acid F
Ethyl myristate	5-acetamido-6-formamido-3-methyluracil	ser-asp
Oxalosuccinic acid	Glutaric acid	cis-6-Dodecen-4-olide
NNN	1-(4,7-Dimethylpyrazolo[5,1-c][1,2,4]triazin-3-yl)ethan-1-one	butanethiol
acetic acid geranyl ester	triacetic acid	Isonicotinylglycine
2-Oxo-4-hydroxy-5-aminovalerate	4-Methyleneglutamic acid	Acetylhistamine
MFCD00059633	5-Deoxy-5-aminoshikimic acid	Artemotil
3-(2-Thienyl)-DL-alanine	6-Amino-9H-purine-9-propanoic acid	3,7,9-Trihydroxy-6H-[1]benzofuro[3,2-c]chromen-6-one
Botrydial	D-Glucopyranuronic acid	2,3-Dihydrothiophene
(10E,15Z)-9,12,13-Trihydroxy-10,15-octadecadienoic acid	beta-D-Xylopyranose	coenzyme Q1
D-Sucrose	2,5-di-tert-Butylhydroquinone	11-Deoxocucurbitacin I
Pyrazinemethanethiol	D-Xylulosonic acid	15,16-DiHODE
2-Amino-5-{[(4,5-dihydroxy-2-cyclopenten-1-yl)amino]methyl}-4H-pyrrolo[2,3-d]pyrimidin-4-on	AMPA	TU4153400
N-(-Ketocaproyl)-L-homoserine lactone	LT9970000	(6Z)-3,6,10-Trimethyl-3a,5,8,10,11,11a-hexahydrocyclodeca[b]furan-2,9(3H,4H)-dione
TI1100000	ON1225000	Palmitic acid
Furan-2-ylmethanethiol	(.+/-.)-Laudanosine	α,α-Trehalose
Phenazopyridine	DL-Mevalonic acid	Quillaic Acid
1,4-Bis(2-ethylhexyl) sulfosuccinate	2-Ethylglutaric acid	12-HSA
MFCD00672375	1,3,7-Trimethyluric acid	epsilon-Caprolactone
6-Methyl-7,8-dihydroimidazo[1,5-c]pyrimidine-5(6H)-thione	shrimp shell	Propapyriogenin A2
N-formylmaleamic acid	Azelaic acid	13(S)-HOTrE
isosorbide dinitrate	Dinoterb	ethionamide S-oxide
Î²-D-Ribofuranosylcreatine	1-Deoxypentitol	MFCD00133847
L-dihydroanticapsin	Lumazine	(4Z)-9,11-Dihydroxy-1,1,4,7a-tetramethyl-10-(3-methylbutanoyl)-1,1a,2,3,6,7,7a,8,13a,13b-decahydrocyclopropa[9,10]cyclodeca[1,2-b]chromene-12-carbaldehyde
5-Chloro-3-fluoro-1,2-benzenediol	Metirosine	Matairesinol
NP-003039	BX9100000	Asiatic acid
S-Allyl propanethioate	2-Cyclopenten-1-one, 4-hydroxy-3-methyl-2-(2-pentenyl)-	8-Methyl-8-azabicyclo[3.2.1]oct-3-yl (3S)-1,2-dithiolane-3-carboxylate
MEIQX	Vitamin C	4-Hydroxyprolylserine
2,4-Dibromophenol	N-Acetyl-6-oxo-L-norleucine	Histidylglycine
Carbimazole	4-[Amino(carboxy)methyl]-5-oxoproline	Pentyl isothiocyanate
(S)-ACPA	cis-2-Carboxycyclohexyl-acetic acid	cadusafos
Chlorambucil	Methional	UNII:V9Q7UUZ15U
felbamate	3,5-Dinitroguaiacol	(1R,5S,6R,7R,9S,11S,12S,13S,14S)-3-Amino-14-methyl-8,10-dioxa-2,4-diazatetracyclo[7.3.1.1~7,11~.0~1,6~]tetradec-2-ene-5,9,12,13,14-pentol
nitrilotriacetic acid	Protirelin	CU5774000
(2α,3β,19α)-2,3,19-Trihydroxyolean-12-en-28-oic acid	NP-002089	2'-Deoxycytidine
Oxaburimamide	1-(Propyldisulfanyl)-1-(propylsulfinyl)propane	Aciclovir
.alpha.-Glucoheptitol	4-Acetamido-2-amino-6-nitrotoluene	N-Acetylcytidine
Isoniazid	2-Oxo-5-pentyltetrahydro-3-furancarboxylic acid	Valproic acid
-	N-Acetyl-D-quinovosamine	L-3-hydroxykynurenine
-	4-hydroxyamino-2,6-dinitrotoluene	9-(beta-D-Arabinofuranosyl)-2-imino-3,7,8,9-tetrahydro-2H-purin-6-ol
-	Nojirimycin	asn-val
-	Methylmalonic acid	Homocysteine thiolactone
-	(1S,3R,4S)-3,4-Dihydroxycyclohexanecarboxylic acid	Ethyl 3-(furfurylthio)propionate
-	N-Acetylneuraminic acid	2-Monocaprin
-	Aceglutamide	Methyl 9-oxononanoate
-	N-Undecanoylglycine	Furagin
-	Citric acid	-
-	2-Methyltetrahydrothiophen-3-one	-
-	Glucoheptonic Acid	-
-	Kinetin	-
-	N-(Carboxymethyl)serine	-
-	N-Acetyl-L-aspartic acid	-
-	MHPG	-
-	(3-Hydroxy-3,4,5,6-tetrahydro-1H-cyclopenta[c]furan-4-yl)acetic acid	-
-	2,4-Dimethylthiazole	-
-	mycinose	-
-	2,4,5-Trimethyl-1,3-thiazole	-
-	Venlafaxine	-
-	Troxacitabine	-
-	2,4-di-tert-Butylphenol	-
-	4-(Nitrosoamino)-1-(3-pyridinyl)-1-butanone	-
-	Glu-Gly	-
-	N.pi.-Methyl-L-histidine	-
-	Butyl isothiocyanate	-
-	1-(5-Azido-5-deoxy-beta-D-ribofuranosyl)-4-hydroxy-2(1H)-pyrimidinone	-
-	Aspartyl-L-proline	-
-	APM	-
-	anticapsin	-
-	2,6-Dideoxy-3-O-methyl-L-arabino-hexopyranose	-
-	Lycocernuine	-
-	Sarin	-
-	(±)12(13)-DiHOME	-
-	Hex-2-ulose	-
-	2-Deoxy-scyllo-inosamine	-
-	Dodecyl sulfate	-
-	Eglumetad	-
-	2,4-DIMETHYL-5-VINYLTHIAZOLE	-
-	4-Dodecylbenzenesulfonic acid	-
-	Thio-THIP	-
-	S-Allyl-L-cysteine	-
-	Roxatidine	-
-	Etilevodopa	-
-	Ethyl 2-(methyldithio)propanoate	-
-	Estrone	-
-	Cytidine	-
-	3-(5-oxoisoxazolin-4-yl)-L-alanine	-
-	3-(1-Piperazinyl)benzisothiazole	-
-	(+/-)-Camphoric acid	-
-	2-thiazolylethylamine	-
-	3,4-dihydroxyglutamic acid	-
-	Ronidazole	-
-	propylthiouracil	-
-	Corn thiazoline	-
-	NX8480000	-
-	Isoflurophate	-
-	Estradiol Dipropionate	-
-	NP-019547	-
-	Phenylmethylsulfonyl fluoride	-
-	Dodecanedioic acid	-
-	Butylparaben	-
-	N-(6-Amino-5-nitro-4-pyrimidinyl)pentofuranosylamine	-
-	(2S,3R,5S)-3-(2-Aminoethyl)-7-oxo-4-oxa-1-azabicyclo[3.2.0]heptane-2-carboxylic acid	-
-	Quisqualic acid	-
-	benthiocarb	-
-	Proxyphylline	-
-	Tetrahydrothiophene	-
-	N-(Carboxymethyl)-Î²-alanine	-
-	methyl thioacetate	-
-	N-methyl-2-oxoglutaramic acid	-
-	Ser-Glu	-
-	Nifuraldezone	-
-	OA-6129 D	-
-	2-Deoxyhexopyranose	-
-	N-Hydroxy-meIQX	-
-	Diethyl (2R,3R)-2-hydroxy-3-methylsuccinate	-
-	(3aS,6S,6aR)-3,3,3a,6-Tetrahydroxytetrahydrofuro[3,2-b]furan-2(3H)-one (non-preferred name)	-
-	NP-020283	-
-	2-Methyl-3-undecyl-1,4-naphthoquinone	-
-	desciclovir	-
-	lactide	-
-	dihydroxyphenylalanine	-
-	Trolox	-
-	(15Z)-9,12,13-Trihydroxy-15-octadecenoic acid	-
-	Ethyl malate	-
-	Hexaric acid	-
-	alpha-ketoadipic acid	-
-	3-Methylsulfolene	-
-	5-Acetyl-2,4-dimethylthiazole	-

-	Penbutolol	-
-	3-(2-Amino-4-pyrimidinyl)alanine	-
-	Citiolone	-
-	N-(4-Hydroxyhexanoyl)glycine	-
-	5,6,7,8-tetrahydropterin-6-carboxylic acid	-
-	2-methylcitric acid	-
-	Amrinone	-
-	5-amino-5-deoxy-3-dehydroshikimic acid	-
-	Mizoribine	-
-	2,2'-Ethylidene-bis(4,6-di-tert-butylphenol)	-
-	Phloionolic acid	-
-	Pidotimod	-
-	(S)-(-)-Goitrin	-
-	MFCD00037215	-
-	NP-011548	-
-	VS1150000	-
-	1-(4-Methoxyphenyl)-3-pentanone	-
-	3-oxopalmitic acid	-
-	Methyldopa	-
-	4-Hydroxy-L-threonine	-
-	NS1800000	-
-	guanitoxin	-
-	MFCD00209536	-
-	N-Acetylglucosaminitol	-
-	cetoniacytone A	-
-	D-(−)-Erythrose	-
-	2,4-Dihydroxy-6-[(2E,6E,10E)-3,7,11,15-tetramethyl-2,6,10,14-hexadecatetraen-1-yl]phenyl acetate	-
-	9,10-Dihydroxystearic acid	-
-	2-Hydroxy-6-oxo-1,6-dihydro-3-pyridinecarboxylic acid	-
-	Sebacic acid	-
-	(E)-Furazolidone	-
-	(2E)-2-Cyano-N-(ethylcarbamoyl)-2-(methoxyimino)acetamide	-
-	3-[(2Z)-1-Oxo-2-buten-2-yl]pentanedioic acid	-
-	Glycyl-4-hydroxyproline	-
-	MFCD00004231	-
-	Diethylpyrocarbonate	-
-	carglumic acid	-
-	Juniperic acid	-
-	Glycylglycylglycine	-
-	6-(1-Hydroxyethyl)-3-(hydroxymethyl)-2,7-dioxabicyclo[4.1.0]hept-3-en-5-one	-
-	Glucuronamide	-
-	8-DEMETHYL MEIQX	-
-	1,3,4,5-Tetrahydroxycyclohexanecarboxylic acid	-
-	glu-ser	-
-	mephenesin	-
-	Azacitidine	-
-	Guaifenesin	-
-	5-[2-(3-Furyl)ethyl]-8a-(hydroxymethyl)-5,6-dimethyl-3,4,4a,5,6,7,8,8a-octahydro-1-naphthalenecarboxylic acid	-
-	2,5-Dimethyl-4-ethoxy-3(2H)-furanone	-
-	glu-asp	-
-	Hept-2-ulose	-
-	XT5075000	-
-	paeonilactone A	-
-	Bufa-20,22-dienolide	-
-	Sulfolane	-
-	3-Thiomorpholinecarboxylic acid	-
-	protionamide	-
-	4,5-Dihydroxy-3-oxo-1-cyclohexene-1-carboxylic acid	-
-	fenfluramine	-
-	XJ5104000	-
-	2-Methoxy-5-nitrophenol	-
-	Diethyl tartrate	-
-	3,4,5-Trihydroxy-1-cyclohexenecarboxylic acid	-
-	Orotic acid	-
-	2,3,4,5-tetrahydrodipicolinic acid	-
-	Sulfurol	-
-	2-(Methylthio)ethanol	-
-	Allyl isothiocyanate	-
-	Methyl bisnorbiotinyl ketone	-
-	(4R,5S,9S,10R,12S,13S)-1,5,9-Trimethyl-11,14,15,16-tetraoxatetracyclo[10.3.1.0~4,13~.0~8,13~]hexadecan-10-[illegible]	-
-	IN00105	-
-	5-Hydantoinpropionic acid	-
-	2-Isobutylthiazole	-
-	(5S)-5-(Carboxymethyl)-L-proline	-
-	Etanidazole	-
-	1,3,4-Trihydroxy-5-oxocyclohexanecarboxylic acid	-
